# Lifestyle, health behavior, and oral health differences among dentistry, nutrition, and fine arts university students

**DOI:** 10.3389/fpubh.2026.1764709

**Published:** 2026-02-20

**Authors:** Irene Coll Campayo, Maria Servera Fuster, Nora López-Safont

**Affiliations:** 1 Faculty of Dentistry, University ADEMA School, Palma, Spain; 2 ADEMA-Health Group, University Institute for Research in Health Sciences (IUNICS), Palma, Spain; 3 Biology Department, University of the Balearic Islands, Palma, Spain

**Keywords:** dietary habits, health behaviors, health promotion, lifestyle, oral health, physical activity, university students

## Abstract

**Background/Objectives:**

University students' lifestyle habits influence their general and oral health in the medium and long term. This study compared physical activity, dietary habits, tobacco and alcohol consumption, and oral hygiene practices among students of Dentistry, Nutrition, and Fine Arts, aiming to identify differences that may guide future preventive interventions.

**Methods:**

A descriptive cross-sectional study was conducted between June and July 2025 at the ADEMA University School (Palma de Mallorca, Spain). The final sample included 165 students (115 Dentistry, 32 Nutrition, and 18 Fine Arts) aged 18–30 years. A validated self-administered questionnaire, adapted from World Health Organization instruments and the ENALIA study, was used to collect sociodemographic variables, physical activity level, screen time, dietary habits, tobacco and alcohol use, and oral hygiene practices. Data were analyzed using descriptive statistics; χ^2^ and ANOVA tests were applied according to variable type, with statistical significance set at *p* < 0.05. The study was approved by the Balearic Islands Research Ethics Committee (IB 5691/24 PI).

**Results:**

Nutrition students exhibited the healthiest profiles: 60% reported ≥2–3 h/week of exercise and higher daily fruit and vegetable intake. Dentistry students showed the best self-perceived oral health and the greatest brushing frequency. Fine Arts students exhibited higher sedentary behavior and poorer dietary and oral hygiene habits. Significant differences among degree programs were found in physical activity, diet, and oral health variables (*p* < 0.05).

**Conclusions:**

Relevant differences in lifestyle and health behaviors were observed according to academic degree. University education appears to influence the adoption of healthy habits; therefore, health promotion programs integrating oral health as an essential component of overall well-being are recommended.

## 1 Introduction

University is a key period for developing lifestyle habits that influence health over time. Throughout these years, young people have gained autonomy in their routines, diet, and self-care, which can promote both the adoption of healthy practices and the persistence of risky behaviors (1, 2). Several studies have shown that university students exhibit various health behaviors depending on their environment, field of study, or gender, making this group a priority target for health promotion. In addition, the transition to university life is often accompanied by an increase in sedentary behavior, dietary changes, and less overall self-care, particularly as it relates to oral and mental health (3, 4).

The main determinants of general and oral health include diet, physical activity, tobacco and alcohol use, and oral hygiene habits (5–8).

Several studies have indicated that health-related behaviors vary according to gender and academic field (2, 9). In particular, students enrolled in health science programs tend to show increased awareness of prevention and healthier practices than those in non-health disciplines (10). This disparity may be influenced by the inclusion of content on well-being and prevention in the curriculum, as well as by the perception of the professional role as a health promoter.

Oral health is an essential component of overall well-being and an important indicator of quality of life (7, 11). Current scientific evidence considers oral health an essential component of overall well-being and a marker of social inequalities in health. The lack of proper hygiene habits, in addition to dietary and behavioral factors, can contribute to pathologies that affect quality of life, even in apparently healthy young people. However, oral health typically receives less attention in university health promotion programs (12, 13). Moreover, oral health is closely related to systemic diseases such as cardiovascular and metabolic diseases, and its deterioration can have physical, psychological, and social repercussions (7, 10). In the local context, recent studies of children and adolescents in Mallorca have underscored the link between lifestyle habits, excess weight, and oral health, highlighting the role of diet, screen time, and physical activity in the development of oral diseases (14–16). However, oral health remains an underrepresented aspect of university health promotion programs, despite its fundamental role in preventing chronic diseases and improving quality of life (16).

In recent years, various national and international institutions have promoted the concept of “Healthy Universities,” encouraging the creation of academic settings that facilitate healthy lifestyles and integrate physical, mental, and social health into the university community (17). In Spain, the Spanish Network of Health Promoting Universities (*Red Española de Universidades Promotoras de Salud*- REUPS) has adopted this approach, including aspects such as balanced nutrition, physical activity, and oral health in its priority lines of action (18).

In this respect, the university provides an ideal setting for promoting healthy habits and integrating oral health into a holistic conception of well-being. Analyzing lifestyle and health behavior differences among students in different degree programs makes it possible to identify specific needs and design more effective and equitable education strategies. This study compares the general and oral health habits of Dentistry, Nutrition, and Fine Arts students to identify distinctive patterns that can inform future preventive interventions in the university setting.

## 2 Materials and methods

### 2.1 Study design

An observational, cross-sectional, descriptive study was conducted to analyze and compare lifestyles, general health habits, and oral health among university students pursuing different undergraduate degrees. The design followed the recommendations of the World Health Organization (WHO) for research on health-related behaviors (19).

### 2.2 Scope and study population

The study was conducted at the ADEMA University School, affiliated with the University of the Balearic Islands (Palma de Mallorca, Spain), during the 2024–2025 academic year. The target population was students enrolled in the Dentistry, Human Nutrition and Dietetics, and Fine Arts degree programs.

### 2.3 Sample and selection criteria

The final sample consisted of 165 students: 115 in Dentistry (69.7%), 32 in Nutrition (19.4%), and 18 in Fine Arts (10.9%). Participants were selected through non-probabilistic sampling, and the following inclusion criteria were applied:

- Be enrolled in one of the three aforementioned degree programs.
- Be between 18 and 30 years of age (the upper age limit was extended to include students older than 30 years, reflecting the increasing presence of non-traditional and mature students in contemporary university populations).
- Agree to participate voluntarily by providing written informed consent.

Questionnaires that were incomplete or contained inconsistent answers were excluded.

### 2.4 Study variables

The variables analyzed were grouped into the following blocks:

- Sociodemographic variables: age (years), gender (male, female, other), degree studied (Dentistry, Nutrition, Fine Arts), academic year, and area of residence (rural, urban, suburban).
- Physical activity and sedentary behavior: average daily hours of physical activity per week, type of exercise, frequency of exercise, active transportation (walking or cycling), and daily screen time (TV, computer, cell phone/tablet) during the week and on weekends.
- Eating and dietary habits: number of meals per day, snacking between meals, frequency of consumption of healthy foods (fruit, raw and cooked vegetables, whole wheat bread, cereals) and unhealthy foods (pastries, snacks, French fries, sugary drinks, and energy drinks), as well as intake of fresh meat, milkshakes, and coffee.
- Oral health: frequency of tooth brushing (times/day), type of toothbrush (manual, electric, interdental), use of dental floss, fluoride toothpaste, and mouthwash; self-perception of dental and gum health (excellent, very good, good, normal, poor, don't know); experience of oral pain in the last 12 months and reason for the last visit to the dentist (pain, conservative treatment, tartar removal, check-up, don't know).

### 2.5 Data collection tool

The information was obtained using a structured, self-administered questionnaire, developed based on WHO guidelines for health surveys (13) and the National Survey on Food Consumption Among Children and Young People (*Encuesta Nacional de Consumo de Alimentos en Población Infantil y Juvenil -* ENALIA) (20).

The questionnaire underwent expert review by specialists in public health and preventive dentistry, followed by pilot testing with 15 students to confirm the clarity and consistency of its items. The internal consistency of the instrument was assessed using Cronbach's alpha coefficient, with a value of 0.81, indicating adequate reliability for use in the university population (21).

Participants completed the questionnaire under the in-person supervision of the research team, ensuring that they understood the questions and that the quality of their responses was high.

### 2.6 Procedure

Data were collected between June and July 2025. The questionnaires were distributed in digital format and completed anonymously and voluntarily. Before participating, students received an information sheet about the study objectives and signed an informed consent form. Data confidentiality was guaranteed in accordance with the General Data Protection Regulation (EU) 2016/679 and Organic Law 3/2018 on Personal Data Protection and Guarantee of Digital Rights.

### 2.7 Statistical analysis

The data analysis was performed using the IBM SPSS Statistics software v.29 (IBM Corp., Armonk, NY, USA). Descriptive statistics (frequencies, percentages, means, and standard deviations) were calculated for all variables. Comparisons between degree programs were performed using the chi-square test (χ^2^) for categorical variables, and Fisher's exact test was applied when expected cell counts were small. Continuous variables were analyzed using one-way ANOVA. Prior to ANOVA, assumptions of normality and homogeneity of variances were assessed using standard diagnostic procedures.

*Post hoc* multiples comparisions for ANOVA were conducted using Bonferroni corrections where appropriate. Effect sizes were calculated to complement p-values, using Cramer's V for categorical variables and eta squared (η^2^) for ANOVA analyses. A statistical significance level of p < 0.05 was established.

### 2.8 Ethical considerations

Ethics approval for the study was granted by the Balearic Islands Research Ethics Committee (CEI-IB), under approval number IB 5691/24 PI. All participants provided their written informed consent prior to inclusion. The research was conducted in accordance with the principles of the Declaration of Helsinki (2024 version) (22) and the Good Clinical Practice Guidelines (23).

## 3 Results

The sample comprised 165 university students from the ADEMA University School (Palma de Mallorca, Spain). The mean age was 24.7 ± 0.35.

The distribution by degree program was as follows: Dentistry (*n* = 115; 69.7%), Human Nutrition and Dietetics (*n* = 32; 19.4%), and Fine Arts (*n* = 18; 10.9%).

In terms of sociodemographic characteristics, most students resided in urban areas (60.3%), followed by rural areas (27.4%), and suburban areas (12.8%). By age, 46.9% were between 20 and 25 years, 18.2% were under 20, 13.4% were between 25 and 30, and 21.9% were over 30. With regard to the academic year, there was a slight predominance of first-year (30.4%) and fourth-year students (23.7%).

In terms of gender, the Dentistry and Nutrition degree programs had similar proportions of women (76.5% and 75%, respectively), while in Fine Arts, the proportion of women was somewhat lower (66.7%). It should be noted that one Fine Arts student identified with the category “other” (0.61% of the total) (Table 1).

**Table 1:** Sociodemographic characteristics of the sample according to degree.

**Variable**		**Dentistry**		**Nutrition**		**Fine Arts**		**Total**	
		***n***	**%**	***n***	**%**	***n***	**%**	***n***	**%**
Sex	Male	27	23.4	8	25.0	5	27.8	40	24.3
	Female	88	76.5	24	75.0	12	66.7	124	75.6
	Other	–	–	–	–	1	5.6	1	0.61
Area of Residence	Rural	27	23.4	11	34.3	7	38.9	45	27.4
	Urban	72	62.6	18	56.2	9	50.0	99	60.3
	Suburban	16	13.9	3	9.4	2	11.1	21	12.8
Age	< 20 years	17	14.7	7	21.8	6	33.3	30	18.2
	20–25 years	56	48.6	14	43.7	7	38.9	77	46.9
	25–30 years	17	14.7	5	15.6	–	–	22	13.4
	>30 years	25	21.7	6	18.7	5	27.8	36	21.9
Year	First	34	29.5	9	28.1	7	38.9	50	30.4
	Second	7	6.1	10	31.2	8	44.4	25	15.2
	Third	15	13.0	6	18.7	2	11.1	23	14.0
	Fourth	31	26.9	7	21.8	1	5.6	39	23.7
	Fifth	28	24.3	–	–	–	–	28	17.0

### 3.1 Physical activity

Nutrition students showed a more active profile: 60% exercised at least 2–3 h per week, and none reported total inactivity. In contrast, Fine Arts students participated less in regular physical activity and spent more time sitting, associated in particular with screen use. Dental students exhibited intermediate behavior, with moderate levels of physical activity (Table 2).

**Table 2:** Mean daily hours of physical activity during weekdays by degree program.

**Degree program**	**Degree**	***n***	**Mean**	**SD**	**SE**	**95% CI**	***p* value**
	Dentistry	115	1.96	1.33	0.12	(1.71–2.21)	0.012
	Nutrition	32	2.68	0.89	0.15	(2.36–3.01)	
	Fine Arts	18	1.88	1.18	0.27	(1.30–2.47)	

*n*, sample size; SD, standard deviation; SE, standard error; CI, confidence interval; one-way ANOVA, *p* < 0.05 and Bonferroni *post hoc* analysis.

### 3.2 Screen use

Fine Arts students recorded the most hours spent in front of screens both during the week and on the weekend, while Nutrition students had the lowest values and Dentistry students had intermediate values (Fine Arts: 1.88 ± 0.29 h/day; Dentistry: 1.55 ± 0.11 h/day; Nutrition: 1.03 ± 0.15 h/day; *p* = 0.028). During the weekend, the pattern was the same, with 2.16 ± 0.27 h/day in Fine Arts, 1.90 ± 0.12 h/day in Dentistry, and 1.28 ± 0.18 h/day in Nutrition (*p* = 0.022) (Table 3).

**Table 3:** Mean daily screen time during weekdays and weekends by degree program.

**Measurement periods**	**Degree**	***n***	**Mean**	**SD**	**SE**	**95% CI**	***p* value**
Screen time during weekdays	Dentistry	115	1.55	1.22	0.11	(1.32–1.78)	0.028
	Nutrition	32	1.03	0.89	0.15	(0.70–1.35)	
	Fine Arts	18	1.88	1.23	0.29	(1.27–2.50)	
Screen time during weekends	Dentistry	115	1.90	1.31	0.12	(1.66–2.14)	0.022
	Nutrition	32	1.28	1.02	0.18	(0.91–1.65)	
	Fine Arts	18	2.16	1.15	0.27	(1.59–2.73)	

*n*, sample size; SD, standard deviation; SE, standard error; CI, confidence interval; one-way ANOVA: *p* < 0.05 and Bonferroni post hoc analysis.

### 3.3 Oral health habits

Oral hygiene practices differed significantly among degree programs. Most students used manual toothbrushes (86.6%), with no significant differences among groups (*p* = 0.726).

The use of electric toothbrushes and interdental brushes was more frequent among Dentistry students, although statistical significance was not reached (*p* > 0.05).

The use of dental floss and fluoride toothpaste was significantly higher in the Dentistry group (p < 0.001), while mouthwash use was similar across degree programs.

Overall, Dentistry students exhibited a more comprehensive oral hygiene pattern, characterized by a greater diversity of tools used (Table 4).

**Table 4:** Oral hygiene practices by degree program

**Variable**		***n***	**%**	**Dentistry**	**Nutrition**	**Fine Arts**	***p* value**
Manual toothbrush	Yes	143	86.6	99	29	15	0.726
	No	22	13.3	16	3	3	
Electric toothbrush	Yes	64	38.7	50	8	6	0.146
	No	101	61.2	65	24	12	
Interdental toothbrush	Yes	49	29.6	38	6	5	0.289
	No	116	70.3	77	26	13	
Dental floss	Yes	120	72.7	95	15	10	< 0.001^*^
	No	45	27.2	20	17	8	
Fluoride toothpaste	Yes	132	80	109	15	8	< 0.001^*^
	No	33	20	6	17	10	
Mouthwash	Yes	55	33.3	37	12	6	0.852
	No	110	66.6	78	20	12	

*n*, number of participants; chi-square: ^*^ variable with significant effect (p < 0.05).

Statistically significant differences were observed among Dentistry, Nutrition, and Fine Arts students in their self-perception of dental and gum health (*p* < 0.001). 68.7% of Dentistry students rated their dental health as “excellent” or “very good,” compared to 40.6% in Nutrition and 22.2% in Fine Arts. Similarly, 67.8% of Dentistry students considered their gum health to be “excellent” or “very good,” compared to 31.3% of Nutrition students and 33.3% of Fine Arts students.

Significant differences were also found in the reason for consultation (*p* < 0.001): Dentistry students most frequently attended for check-ups or tartar removal (76.5%), whereas among Fine Arts students, consultations for pain or conservative treatment predominated (38.9%). Within the Nutrition group, both types of reasons were represented in comparable proportions (around 22%) (Table 5).

**Table 5:** Self-perception of oral health and reasons for consultation according to degree program.

**Variable**		***n***	**%**	**Dentistry**	**Nutrition**	**Fine Arts**	***p* value**
Dental Health	Excellent	29	17.5	27	1	1	< 0.001^*^
	Very good	67	40.6	52	12	3	
	Good	45	27.2	29	9	7	
	Normal	16	9.69	5	6	5	
	Bad	7	4.24	1	4	2	
	Don't know	1	0.60	1	–	–	
Gum Health	Excellent	33	21.1	29	4	–	< 0.001^*^
	Very good	61	39.1	49	6	6	
	Good	36	23.0	22	8	6	
	Normal	18	11.5	3	12	3	
	Bad	6	3.84	1	–	–	
	Don't know	1	0.64	1	–	–	
Reason for Consultation	Pain	23	13.9	14	3	6	< 0.001^*^
	Conserv. Treat.	16	9.69	11	4	1	
	Scaling	44	26.6	44	–	–	
	Check-up	80	48.4	44	25	11	
	Don't know	2	1.21	2	-	-	

*n*, number of participants; %: percentage of responses within each group; chi-square: ^*^variable with significant effect (*p* < 0.05).

Regarding the experience of oral pain or discomfort in the last 12 months, significant differences were observed among degree programs (*p* = 0.021). 24.5**%** of students reported having experienced oral pain in the last year. The proportion was highest in Nutrition (37.5%) and Fine Arts (41.2%), while it was 18.3% in Dentistry. In contrast, 75.4% of the sample reported no oral pain during the same period, with this outcome observed predominantly in Dentistry (81.7%). These results reflect that Dentistry students, who have better hygiene habits and more frequent preventive checkups, report a lower prevalence of oral pain compared to Nutrition and Fine Arts students (Table 6).

**Table 6:** Oral pain by degree program.

**Variable**		***n***	**%**	**Dentistry**	**Nutrition**	**Fine Arts**	***p* value**
Oral pain in the last 12 months	Yes	40	24.5	21	12	7	0.021 ^*^
	No	123	75.4	93	20	10	

*n*, sample, yes, no; chi-square ^*^ variable with significance (*p* < 0.05).

### 3.4 Frequency of food consumption

Regarding the structure of daily meals, most participants reported eating three meals a day (58.7%), with significant differences among degree programs (*p* = 0.047).

Nutrition students were the most likely to eat more than three meals a day, while Dentistry students had a higher proportion of participants who consumed two or fewer meals a day.

Snacking between meals was a common habit in all the groups (≈50%), with no statistically significant differences (Table 7).

**Table 7:** Frequency of daily meals and snacking according to degree program.

**Variable**		***n***	**%**	**Dentistry**	**Nutrition**	**Fine Arts**	***p* value**
Meals per day	Two or fewer	25	15.1	19	3	3	0.047^*^
	Three	97	58.7	68	15	14	
	More than three	43	26.0	28	14	1	
Snacks	Yes	83	50.3	63	12	8	0.195
	No	82	49.6	52	20	10	

*n*, sample; chi-square: ^*^ variable with significant effect (*p* < 0.05).

A detailed analysis of food consumption showed a healthier pattern among Nutrition students, with a higher frequency of consumption of whole wheat bread, fruits, and vegetables (*p* < 0.05).

Conversely, Fine Arts students reported lower consumption of fruits and vegetables and higher consumption of pastries and snacks, although this was not statistically significant. The consumption of white bread, cereals, coffee, and energy drinks did not show significant differences among degree programs (*p* > 0.05) (Table 8).

**Table 8:** Frequency of consumption.

**Food item**		***n***	**%**	**Dentistry**	**Nutrition**	**Fine Arts**	***p* value**
White bread	Monthly	97	58.7	66	24	7	0.140
	Several times a week	42	25.4	31	5	6	
	Once or several times a day	26	15.7	18	3	5	
Whole wheat bread	Monthly	76	46	56	11	9	< 0.001^*^
	Several times a week	54	32.7	42	5	7	
	Once or several times a day	35	21.2	17	16	2	
Cereals	Monthly	132	80	93	26	13	0.188
	Several times a week	20	12.1	15	4	1	
	Once or several times a day	13	7.87	7	2	4	
Pastries	Monthly	140	84.8	98	29	13	0.251
	Several times a week	19	11.5	14	1	4	
	Once or several times a day	6	3.63	3	2	1	
French fries	Monthly	149	90.3	103	30	16	0.603
	Several times a week	14	8.48	11	1	2	
	Once or several times a day	2	1.21	1	1	-	
Vegetables/salad	Monthly	30	18.1	23	6	1	0.010^*^
	Several times a week	65	39.3	52	5	8	
	Once or several times a day	70	42.4	40	21	9	
Cooked vegetables	Monthly	44	26.6	36	3	5	0.005^*^
	Several times a week	71	43	53	11	7	
	Once or several times a day	50	30.3	26	18	6	
Fruit/juice	Monthly	22	13.3	18	2	2	0.004^*^
	Several times a week	59	35.7	48	4	7	
	Once or several times a day	84	50.9	49	26	9	
Fresh meat	Monthly	29	17.5	26	3	–	0.014^*^
	Several times a week	85	51.5	51	19	15	
	Once or several times a day	51	30.9	38	10	3	
Milkshakes	Monthly	144	87.2	99	28	17	0.405
	Several times a week	17	10.3	14	2	1	
	Once or several times a day	4	2.42	2	2	–	
Energy drinks	Monthly	146	88.4	102	30	14	0.222
	Several times a week	17	10.3	12	1	4	
	Once or several times a day	2	1.21	1	1	0	
Coffee	Monthly	60	36.3	43	12	5	0.515
	Several times a week	30	18.1	22	3	5	
	Once or several times a day	75	45.4	50	17	8	

*n*, sample; chi-square: ^*^variable with significant effect (*p* < 0.05).

## 4 Discussion

This study analyzed the lifestyles, health habits, and oral health status of university students in different degree programs at the ADEMA University School, identifying significant differences according to academic field. The results indicate healthier profiles among students enrolled in Nutrition and Dentistry compared to those in Fine Arts. This pattern mirrors finding from previous studies, showing that students in Health Sciences tend to exhibit greater health awareness and more favorable lifestyle behaviors than those in non-health disciplines (2, 24).

Although the present study was conducted in a specific geographical and sociocultural context (Mallorca, Spain), similar patterns have been reported in university populations from other national and international settings (25). Evidence from both European and non-European contexts consistently indicates that Health Sciences students adopt healthier lifestyle behaviors and demonstrate greater preventive awareness than their peers in non-health-related fields (2, 24). This consistency across settings suggests that differences in health-related behaviors are consistently associated with academic field across diverse settings. However, several authors caution that this advantage may decrease as the program progresses, especially during periods of high academic workload or stress, reinforcing the need to maintain ongoing health promotion programs in the setting (10, 26). Given the cross-sectional design of the study, these findings should be interpreted as associations rather than causal relationships.

Regarding physical activity, Nutrition students were the most active, while Fine Arts students were the least active, mainly due to spending more time sitting in front of screens. These results are consistent with recent studies showing associations between intrinsic motivation, academic context (27) and sports participation among university students (1, 2, 5). It has also been observed that physically active students have better psychological well-being (24). Physical inactivity has been identified as one of the main risk factors for non-communicable diseases in Spain (28). The nature of art studies, involving long hours of sitting and using digital devices, may be associated with lower levels of physical activity. Protracted periods of sitting are associated with an increased risk of obesity, metabolic syndrome, and poorer oral health (14–16). Recent studies conducted among Spanish university students during and after the COVID-19 lockdown have revealed an increase in sedentary behavior and a decrease in physical activity, particularly among women (29). This phenomenon appears to be linked to the digitization of education and shifting patterns of youth leisure activities. The WHO emphasizes that regular physical activity, at least 150 min per week of moderate intensity, is essential not only for physical health, but also for mental and social well-being (30).

In terms of oral health, Dentistry students showed a more positive perception of their oral health, brushed more frequently, and used complementary instruments more regularly (7). The more favorable oral health behaviors and perceptions observed among Dentistry students in this study are consistent with findings reported in previous research conducted in dental student populations. Several studies have documented that dental undergraduates generally report better oral hygiene practices, healthier dietary habits, and higher levels of preventive awareness compared with students fron non-dental disciplines, regardless of the stage of training (31, 32). Similar associations between diet, lifestyle factors, and oral health outcomes have also been described in studies incorporating objective clinical indicators such as the DMFT index and periodontal status (33, 34).

However, it is important to note that most of these studies included objective clinical assessments, allowing a more comprehensive evaluation of oral health status. In contrast, the present study focused on self-perceived oral health, which may lead to an overestimation of oral health status among Dentistry students due to higher health literacy. Despite this limitation, perceived oral health remains a relevant indicator, as it is closely associated with health behaviors, quality of life, and patterns of dental care utilization.

These results align with studies on children and adolescents in Mallorca, which show how lifestyle habits, diet, and consumption of ultra-processed foods directly influence oral and periodontal health (14–16). This underscores the importance of incorporating oral health into lifelong health promotion strategies. This relationship between diet and oral health has been widely documented. A diet rich in fruits, vegetables, and fresh foods is associated with a lower risk of tooth decay and periodontal disease, while regular consumption of free sugars and ultra-processed foods increases the risk of oral dysbiosis (35, 36). In addition, recent studies emphasize the importance of considering oral health as an early indicator of overall well-being and health inequalities. In this regard, Nutrition students showed greater adherence to balanced diets, with high consumption of fruits, vegetables, and whole grains, and low consumption of ultra-processed products (37). These findings are in line with previous studies emphasizing that nutrition training fosters the adoption of healthier eating habits, notably the Mediterranean diet (38). Fine Arts students showed a lower frequency of fruit and vegetable intake and higher consumption of pastries and snacks, which could be related to a lack of specific training in health and nutrition. Adherence to the Mediterranean diet has been associated with better academic performance and a lower risk of metabolic diseases in young university students (39). However, inequalities in access to fresh foods and the impact of food marketing can make it difficult to adopt this dietary pattern, especially for students with less knowledge or fewer economic resources (37).

Overall the findings support the existence of meaningful associations between academic field and health-related behaviors, likely reflecting differences in training content, academic culture, and health awareness as well the influence of curriculum and the expectations within each academic program. The university is a strategic setting to implement comprehensive health promotion programs that include oral health as an integral part of overall health (10, 11). This approach is in line with the Health Promoting Universities initiative, promoted by the WHO and the REUPS, which proposes a comprehensive model of university well-being. Incorporating oral health into these strategies makes it possible to advance toward a more holistic view of health and toward university education geared toward sustainability and equity (17, 18).

### 4.1 Strengths and limitations

A major strength of this study is its multidimensional approach, integrating lifestyle behaviors, dietary habits, physical activity, sedentary behavior, and oral health variables. This provides a comprehensive overview of health-related behaviors among university students. The comparison across different academic disciplines offers valuable insight into the influence of educational context on health and preventive behaviors.

Nevertheless, several limitations should be acknowledged. The non-probabilistic sampling design and the unequal distribution of participants across degree programs—reflecting the actual enrollment structure of the institution—may limit the generalizability of the findings and reduce the statistical power of intergroup comparisons, particularly for the Fine Arts group.

Potential confounding variables, including socioeconomic status, academic workload, and prior health education, were not controlled for in this study. These factors may partially explain the observed differences in health behaviors and oral health outcomes between degree programs and should be considered in future research to clarify their influence.

Additionally, medication intake and previous medical conditions were not considered as exclusion criteria and may have influenced self-reported health behaviors or oral health perception. The absence of objective clinical oral health assessments represents another important limitation, as the results rely on self-perceived oral health, which may be affected by reporting bias and differences in health literacy, especially among Dentistry students. In particular, the use of self-reported data may have introduced recall bias or social desirability bias, potentially leading to overestimation of favorable behaviors. Future studies incorporating clinical indicators and objective behavioral measures would allow a more robust assessment of the relationship between lifestyle factors and oral health outcomes.

### 4.2 Implications for practice

The results underscore the need to integrate health education into university curricula, particularly in non-health-related programs, promoting self-care and prevention habits from an educational perspective. The development of health-promoting universities, following the REUPS and WHO guidelines, is an opportunity to strengthen comprehensive health and the sustainability of healthy lifestyles among young people (9, 13).

It is suggested that specific modules on oral health, nutrition, and physical activity be incorporated into Fine Arts programs and other non-health disciplines. In addition, promoting awareness campaigns on sedentary lifestyles, screen exposure, and consumption of ultra-processed products could improve the overall and oral health of the university population.

## 5 Conclusions

The study reveals significant differences in lifestyles and health habits among university students in different degree programs. Nutrition students exhibited the healthiest behaviors, especially in terms of physical activity and diet, while Dentistry students stood out for their better oral health and greater preventive awareness. In contrast, Fine Arts students exhibited more sedentary behaviors and less adherence to healthy habits.

These findings confirm that the academic setting influences the adoption of healthy behaviors, and they underscore the need to develop comprehensive university health promotion programs that include oral health as an essential component of overall well-being.

## Data Availability

The raw data supporting the conclusions of this article will be made available by the authors, without undue reservation.
